# Horizontal Body Position Reduces Cortical Pain-Related Processing: Evidence from Late ERPs

**DOI:** 10.1371/journal.pone.0081964

**Published:** 2013-11-20

**Authors:** Francesca Fardo, Chiara Spironelli, Alessandro Angrilli

**Affiliations:** 1 Department of General Psychology, University of Padova, Padova, Italy; 2 CNR Institute of Neuroscience, Padova, Italy; University of Bologna, Italy

## Abstract

The present study investigated the influence of short-term horizontal body position on pain-related somatosensory processing, by measuring subjective and cortical responses to electrical pain stimulation. Twenty-eight healthy women were randomly assigned to either the experimental horizontal group (Bed Rest, BR) or to the sitting control group (Sitting Control, SC). After 90 minutes in either horizontal or sitting position, the individual pain thresholds were assessed and EEG/self-evaluations recorded during the administration of 180 stimuli delivered to the left forearm. Electrical pain stimuli, calibrated to subjects’ individual pain thresholds, consisted of two different intensity levels: no pain (40% below pain threshold) and pain (40% above pain threshold). Compared with control, BR condition significantly inhibited subjective sensitivity to painless stimuli, whereas electrophysiological results pointed to a reduced slow cortical wave (interval: 300-600 ms) at all stimulus intensities, and smaller amplitude in BR’s right vs. left prefrontal sites. sLORETA analysis revealed that cortical responses were associated with a decreased activation of superior frontal gyrus and anterior cingulate cortex (BA 6/24). Interestingly, BR group only showed significant negative correlations between self-evaluation of painful intensities and frontal cortical negativity, revealing increasingly differentiated responses in bed rest: indeed those BR participants who reported lower pain ratings, displayed reduced negativity within anterior regions. Taken together, results indicate that short-term horizontal position is able to inhibit a fronto-parietal pain network, particularly at the level of central prefrontal regions typically involved in cognitive, affective and motor aspects of pain processing.

## Introduction

The mechanisms supporting pain experience implicate embodied sensory-motor and cognitive factors including physiological processes (e.g., blood pressure and temperature), perceptual discrimination (e.g., spatial, intensity and quality features), and higher order cognitive functions (e.g., attentional and emotional processing) [1-5]. Electrophysiological studies identified the typical components elicited by painful and non-painful electrical stimulation in early-evoked potentials with peak latencies ranging between 40 and 80 ms (P1 and N1), followed by late cortical potentials with latencies from 80-100 to 700 ms [6,7]. In particular, late potentials consist of three components, i.e., a negative peak (N2), a positive peak (P2) and a long-latency positive wave ranging between 300 and 700 ms, with the amplitude maximum over the vertex. Whereas early-evoked potentials reflect the sensory and discriminative analysis of electrical stimulation, late components are supposed to reflect the integration of sensory features with emotional and cognitive aspects of pain processing [2]. Indeed, a long latency posterior positivity has been found when the experimental task requires to discriminate or to evaluate unpredictable pain stimuli of different intensities [8]. Interestingly, in different experimental contexts, the late positive component is modulated by greater processing of biologically relevant emotional stimuli, particularly with negative contents [9], in women more than in men [10], and by anxiety levels [11]. Direct intracranial recordings suggest that the cortical generators of very early components are located in somatosensory associative areas, parietal operculum and insula [12]. Sources of the late N2 component were identified in medial prefrontal and primary somatosensory cortices, whereas the generators of the late positive potentials (i.e., P2 and P3a) have been found in anterior cingulate cortex, but also within frontal, temporal, and parietal associative areas [12].

 Among the conditions involved in pain modulation, body position plays an important role, but has received little attention, so far, as compared with cognitive and emotional variables. An interesting effective manipulation of postural pain alteration is Head Down Bed Rest (HDBR), in which the body is tilted down by 6 degrees. This condition is also termed “simulated microgravity” as it mimics the perceptual and physiological effects of weightless experienced by astronauts during spaceflight. HDBR has been shown to inhibit cortical activity through an increase of the slow frequency EEG delta and theta bands [13,14]. In addition, HDBR was associated with both impaired brain plasticity, as measured by startle reflex habituation [15], and reduced pain perception and cortical pain responses elicited by electrical stimulation [16]. In particular, both early Somatosensory Evoked Potentials (P1) reflecting stimulus physical features, and late potentials (N1 and P2), associated with multimodal integration of sensory, cognitive, and affective pain-related information, were altered in young participants submitted to HDBR [16]. The variety of past results can be coherently interpreted by putting forward the simplest explanation that HDBR is able to inhibit cortical arousal (including cortical-related pain responses), through a still not clarified bottom (body)-up (brain) physiological mechanism.

A similar, but less extreme, condition is the horizontal Bed Rest (BR) which corresponds to the supine position. This represents a more ecological condition, equivalent to that held for long times by bedridden hospitalized patients. Establishing the influence of this body position on pain might be important for the clinical practice, for instance in medical diagnosis based on pain-related symptoms which, if delayed, could have fatal consequences for patients (e.g., in case of medical complications such as an internal hemorrhagic lesion). The present study was aimed at investigating the effects of BR on pain-related responses elicited by electrical tactile stimulation. We aimed at establishing to what extent pain inhibition induced by HDBR position also occurs in the BR position, by analyzing self-evaluations and somatosensory ERPs collected in two groups of participants (i.e., BR group and Sitting Controls). In addition, we aimed to clarify the functional meaning of the observed electrophysiological effects through the estimation of the main cortical generators by sLORETA, and the correlations between subjective and cortical responses. In line with our previous study on HDBR [16], we expected decreased pain sensitivity and cortical processing in BR participants compared with controls. Since it is known from past literature that there are sex differences in pain sensitivity (see for a review 17,18), in order to limit gender-related variance increase in physiological responses, only women were included in the present study.

## Methods

### Participants

 A total of 32 healthy female volunteers were recruited from the University of Padova and randomly assigned to the experimental (i.e., Bed Rest, BR) or control condition (i.e., Sitting Control, SC). Inclusion criteria required that participants did not suffer from chronic pain diseases or other important medical pathologies, and had not consumed drugs or alcohol within three days from the experiment. Every subject received a course credit for participating in the experiment. Four participants, two from each group, were excluded from study analysis because of excessive data artifact. Thus, the final sample consisted of 28 participants, randomly assigned to either the experimental BR (n = 14) or the control SC (n = 14) condition. Groups had similar age (BR mean: 23.14 ± 1.83; SC mean: 22.43 ± 0.65 years; t(1,26) = -1.37, n.s.), state-anxiety (BR mean: 37.71 ± 11.27; SC mean: 37.36 ± 8.63 points; t(1,26) = -0.09, n.s.) and trait-anxiety levels (BR mean: 36.36 ± 4.84; SC mean: 37.29 ± 5.61 points; t(1,26) = 0.47, n.s.). Participants were on average 90% right-handed, according to the Edinburgh Handedness Inventory [19]; had normal or corrected to normal vision and were naïve about the purpose of the experiment. In accordance with the Declaration of Helsinki, every participant gave her written informed consent to the study, which was approved by the Ethics Committee of the Department of General Psychology, University of Padova (Italy).

### Stimuli, Task and Procedure

After participants were randomly assigned to the BR or SC condition, they were prepared for electrophysiological recording. Throughout the experiment, students laid on a mattress parallel to the floor (experimental BR position) or sat on a soft chair (control SC position). A PC laptop screen was firmly placed 50 cm in front of subject’s eyes, to collect pain evaluations. After 90 minutes of rest, during which participants received experimental instructions and were engaged in filler tasks, the pain session started with the assessment of participant’s pain threshold. This was achieved with a method derived from the adaptive procedure, the simple up-down staircase [20,21] which tracks the 50% of the psychometric function. This method has been used in past studies [16,22,23] and the slow random increase of stimulation allows subject to acquaint with the electric procedure, thus providing more reliable and stable thresholds with respect to fast methods based on a few stimulations. However, a drawback of using many stimuli (including the ERP recording phase delivering 60 stimuli for each condition) is that pain sensitivity is subject to a relatively larger habituation. The pain threshold detection phase was guided by a LabVIEW (National Instruments, TX) *ad hoc* program implemented by one author (AA), which controlled electrical stimulation through a parallel port. Electrical stimuli were administered to the left forearm by two surface 10 mm gold electrodes and were delivered by a battery powered, optoisolated, constant current stimulator (with max stimulation level fixed at 10 mA) controlled by PC through parallel port. Electrical pulse lasted 10 ms and the session started with a weak fixed intensity (39 microAmperes, µA), typically undetected by participants. Next, stimulus intensity progressively increased with current increments randomly ranging between 39 and 234 µA. Participants had to evaluate each electric pulse using a visuo-analogue scale (range = 0-10) representing different levels of pain intensities: the critical subjective level to be determined was 5, corresponding to “I start to feel pain”. The interval between the end of one evaluation and the beginning of the next one randomly varied between 3 and 4 seconds. The procedure stopped as soon as the established subjective pain threshold was reached, namely when the mean evaluation of three consecutive electric pulses surpassed the level of 5. After the last pain evaluation, the program computed an on-line regression coefficient between the last seven electrical currents and the corresponding subjective evaluations, and interpolated the exact current intensity (in µA) corresponding to the subjective pain threshold, *a priori* set to 5.

After pain threshold assessment, participants began the experimental task consisting of EEG recording plus subjective pain evaluation during the administration of a series of 180 electrical stimuli. Starting from subjects' individual pain thresholds, three different levels of electrical intensities were administered. The program generated, randomly interspersed: (1) sixty under-threshold electrical pulses, corresponding to -40% pain electrical threshold level (2), sixty electrical pulses at pain threshold level, and (3) sixty over-threshold electrical pulses, corresponding to +40% pain electrical threshold level., Subjects were not made aware that stimuli were of three different intensities. As for pain threshold assessment, each electrical pulse lasted 10 ms and the inter-trial interval randomly varied between 3 and 4 seconds, and soon after the delivery of each stimulus, subjects evaluated the perceived pain level.

### Data recording and analysis

EEG cortical activity was recorded by means of 38 tin electrodes, 31 placed on an elastic cap (Electrocap) according to the International 10-20 system [24], and the remaining 7 electrodes applied below each eye (Io1, Io2), on the two external canthi (F9, F10), nasion (Nz) and mastoids (M1, M2). Cz was used as an on-line recording reference for all channels. Amplitude resolution was 0.1 μV; bandwidth ranged from DC to 100 Hz (6 dB/octave). Sampling rate was set at 500 Hz and impedance was kept below 5 KΩ. EEG was continuously recorded in DC mode and stored for following analysis using the acquisition software NeuroScan version 4.1. Data were off-line re-referenced to the average reference and epoched into 1.2-s intervals, divided into 200 ms before and 1 s after stimulus onset. A 100-ms baseline preceding electric pulse was subtracted from the whole trial epoch. Single trials were corrected for eye movement artifacts, i.e., vertical, horizontal movements and blinking. BESA software (Brain Electrical Source Analysis, 5.1 version) was used to compute ocular correction coefficients, according to Berg and Scherg [25,26]. Each trial was then visually inspected in order to reject any residual artifacts: overall, 12.4% of trails were rejected. 

After visual inspection of grand-average waveforms, EEG data analysis was carried out on the N2 component, between 185 and 215 ms, and a late positive component (i.e., the LPP), between 300 and 600 ms. These relatively late components were sufficiently spread over large areas to allow electrode clustering and detecting more reliable effects. Electrodes were clustered into four regions of interest to perform statistical analysis with two spatial factors of two levels each: Caudality and Laterality. Clusters comprised the average activity of four electrodes and were labeled Anterior Left (AL: IO1, FP1, F7, F9), Anterior Right (AR: IO2, FP2, F8, F10), Posterior Left (PL: CP3, P3, P7, O1), Posterior Right (PR: CP4, P4, P8, O2). 

Since preliminary analysis carried out on subjective pain judgments revealed that all conditions were overall under-estimated, and only over-threshold condition stimuli were evaluated as painful, with average rating around 5 in the 0-10 visuo-analogue scale (corresponding to “I start to feel pain”), we decided to consider, for statistical analyses, only under- (no-pain) and over-threshold (pain) conditions. Thus, subjective pain judgments and electrophysiological components were analyzed by means of analysis of variance (ANOVA), including a between-subjects Group factor (two levels: BR vs. SC position) and a within-subjects Intensity factor (two levels: no-pain vs. pain).

Furthermore, for electrophysiological analyses only, two within-subjects factors were added: Caudality (two levels: anterior vs. posterior) and Laterality (two levels: left vs. right). Post-hoc comparisons were computed using the Newman-Keuls test, and statistical significance was expressed at the p < 0.05 level.

Source localization was carried out by means of standardized Low-Resolution Brain Electromagnetic Tomography (sLORETA; [27]) to identify the neural generators of cortical activity measured in the time interval of interest (i.e., 300-600 ms). Since sLORETA computes the smoothest possible 3D-distributed current source density solution constrained to grey matter, this approach is particularly suited for our analysis since, due to the smoothness constraint, it does not need an *a priori* number of known sources. As a counterpart, sLORETA statistically locates only the main generator of the maximum EEG/ERP component within a specific interval. This does not exclude the co-existence of other generators (which, in experiments like this are typically many), but the tool highlights only the main source among the many activated in a specific interval. Thus, the regions with largest cerebral activation were analyzed in SC compared with BR participants by performing separated two-tailed *t* tests between ERP responses corresponding to each pain intensity (no-pain and pain conditions). A positive *t* value points to a significantly greater activation of SC participants with respect to BR group, whereas a negative *t* value indicates a significantly greater activation of BR vs. SC students. All source location results are expressed in Talairach coordinates [28]. 

As a final step, to clarify the functional meaning of cortical activity occurring in the time window from 300 to 600 ms, Pearson’s correlation analyses were carried out between mean self-evaluations and mean ERP amplitudes of pain condition on those clusters in correspondence of which significant sLORETA effects have been found.

## Results

### Behavioral and electrical threshold data

A Student’s *t* test was carried out to evaluate whether the two groups differed in the electrical pain threshold: according to the main hypothesis, greater current levels could be expected in the BR group. Analysis revealed no between-groups differences (t(1,26) = -0.73, n.s.), as BR and SC participants revealed similar electrical thresholds (3.51 mA ± 2.19 and 2.89 mA ± 2.34, respectively).

**Figure 1:**
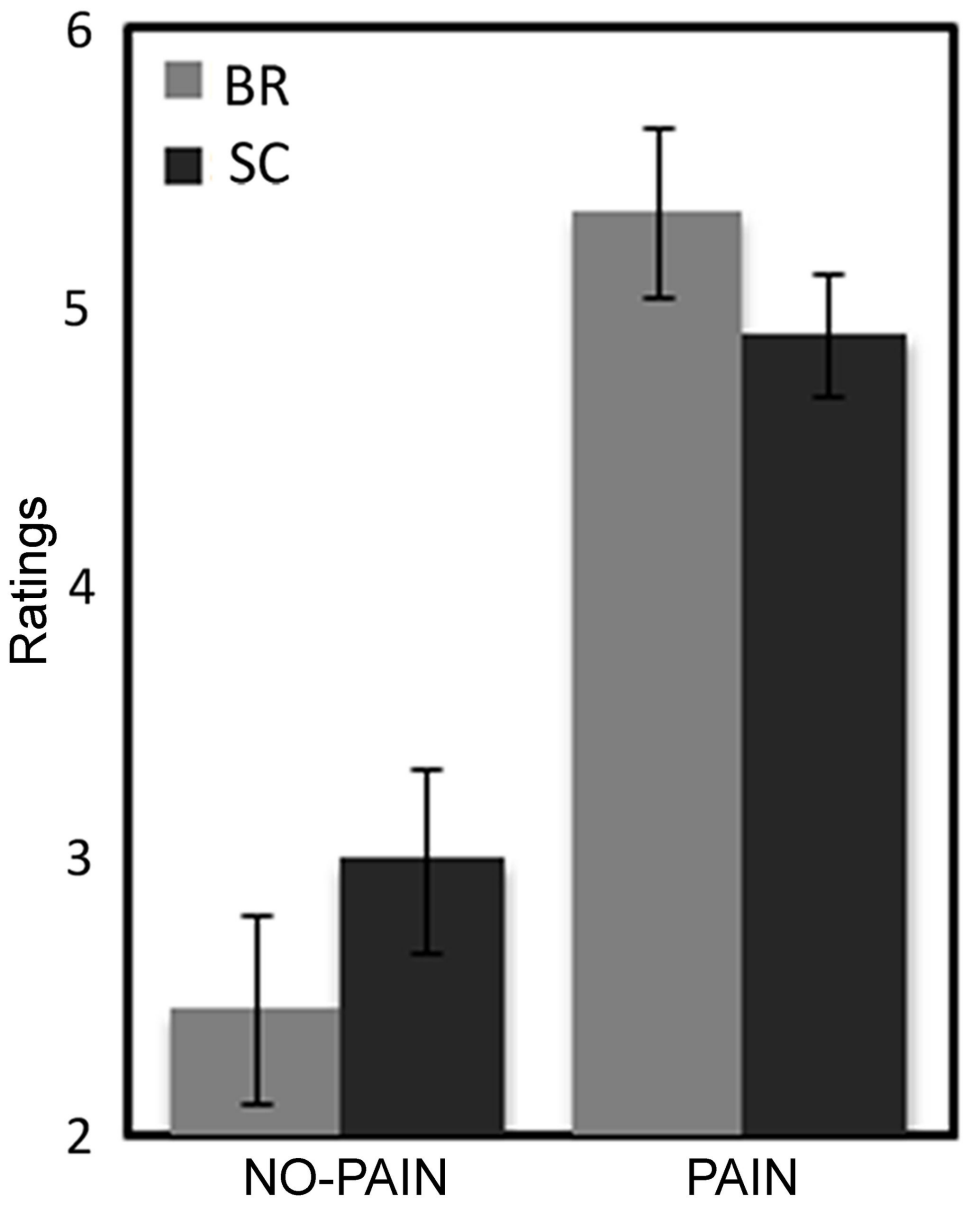
Analysis of subjective pain evaluation: significant two-way Group (BR vs. SC) by Intensity (no-pain vs. pain) interaction. Mean ratings and Standard Error (SE) are depicted for Bed Rest group (grey bars) and Controls (black bars).

ANOVA computed on subjective pain evaluation collected during the EEG recording task revealed a main effect of the Intensity factor (F (1,26) = 159.64, p < 0.001) and a significant Group by Intensity interaction (F (1,26) = 6.72, p < 0.01; Figure 1). Both groups showed low pain ratings during the no-pain condition and higher ratings to pain condition (all p < 0.001). However, compared with controls, BR participants evaluated no-pain stimuli as less intense (p = 0.01). No between-groups differences in subjective evaluations were found for pain condition (Figure 1).

### Electrophysiological data

**Figure 2:**
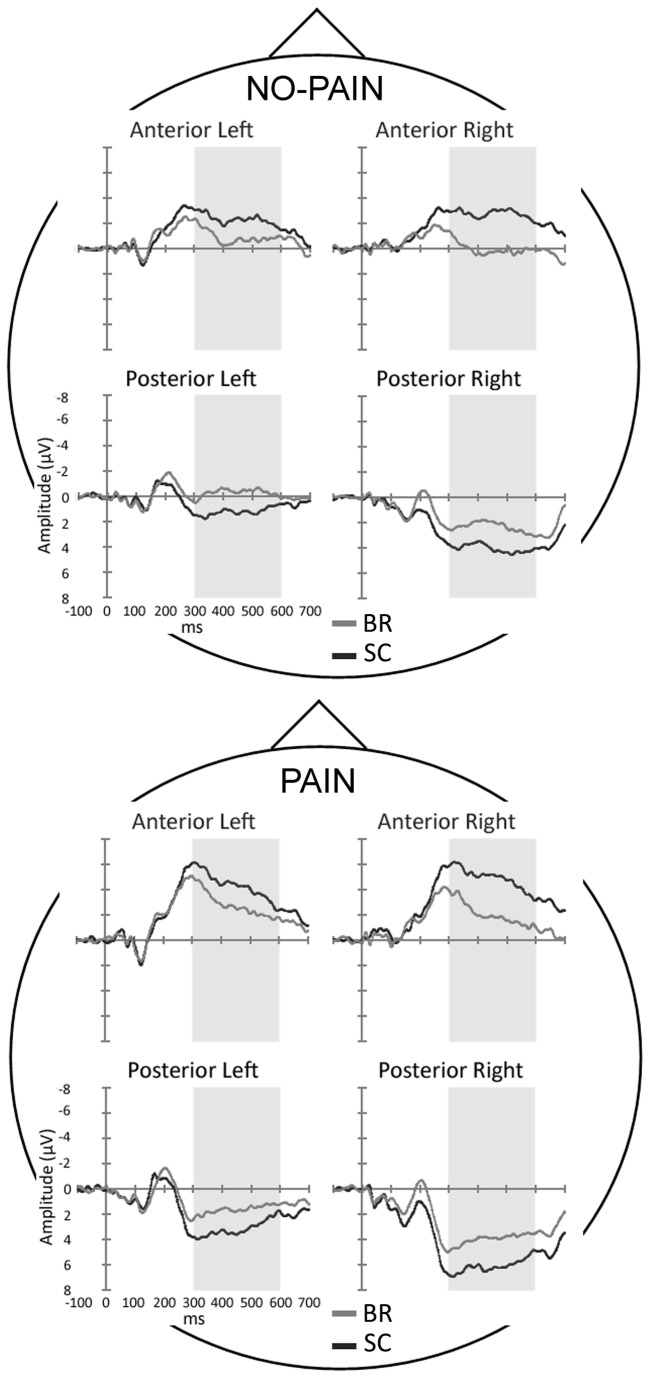
ERP waveforms from the four clusters of electrodes, including the experimental BR (grey line) and the SC control group (black line), in the no-pain and pain conditions. Time-scale is from -100 to 700 ms. Negativity is displayed upward.

**Figure 3:**
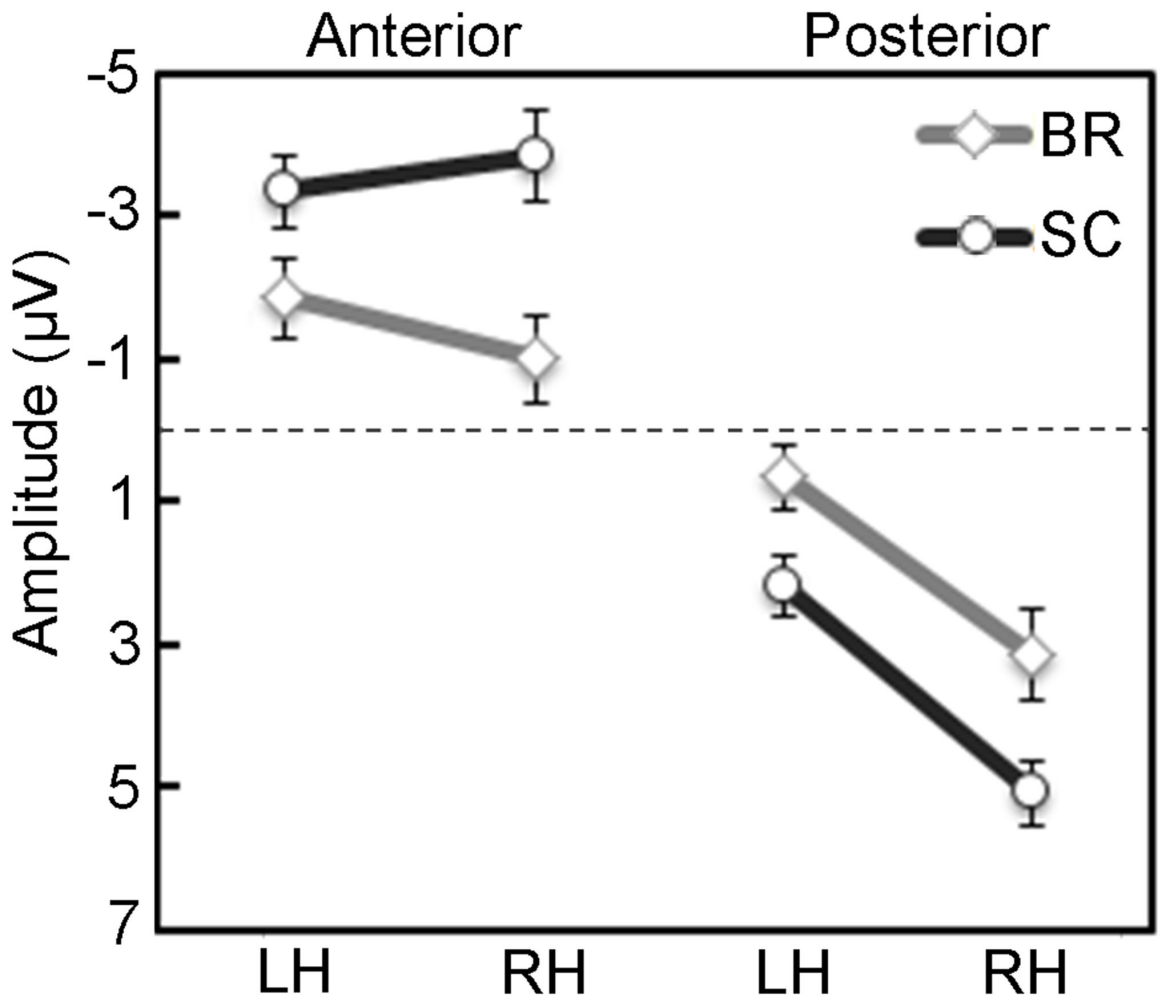
Analysis of the late potentials measured in the 300- to 600-ms epoch after electrical stimuli: significant three-way Group (BR vs. SC) by Caudality (Anterior vs. Posterior regions) by Laterality interaction (Left vs. Right Hemisphere; LH vs. RH). Mean activity and Standard Error (SE) are depicted for Bed Rest group (grey lines and bars) and Sitting Controls (black lines and bars).

Two ANOVAs were carried out on ERPs in the 185- to 215-ms and 300- to 600-ms time windows. The ANOVA centered on the N2 peak revealed no significant main effects or interactions (e.g., Group main effect (F (1,26) = 1.58, ns); three-way Group by Caudality by Laterality interaction (F (1,26) = 1.65, ns). Instead, the analysis of the LPP component yielded significant main effects of Caudality (F (1,26) = 61.92, p < 0.001) and Laterality factors (F (1,26) = 33.50, p < 0.001), which revealed greater positivity in posterior (2.76 µV) with respect to anterior sites (-2.51 µV), and greater positivity in right with respect to left sites (0.83 vs. -0.59 µV, respectively). Finally, the significant three-way Group by Caudality by Laterality interaction (F (1,26) = 5.87, p < 0.05) showed that there were systematic between-groups differences in both anterior and posterior regions (Figures 2 and 3). Late potentials evoked in the BR group showed smaller negativity at anterior sites and smaller positivity at posterior sites with respect to the SC group (p < 0.001), regardless of stimulus intensity. Furthermore, at anterior sites, controls exhibited a bilateral activation, whereas BR participants showed greater negativity on left vs. right locations (p < 0.05). Concerning posterior clusters, greater positivity was found in right compared with left electrodes in both groups (all p < 0.001; Figures 2 and 3). 

### Source analysis

**Figure 4:**
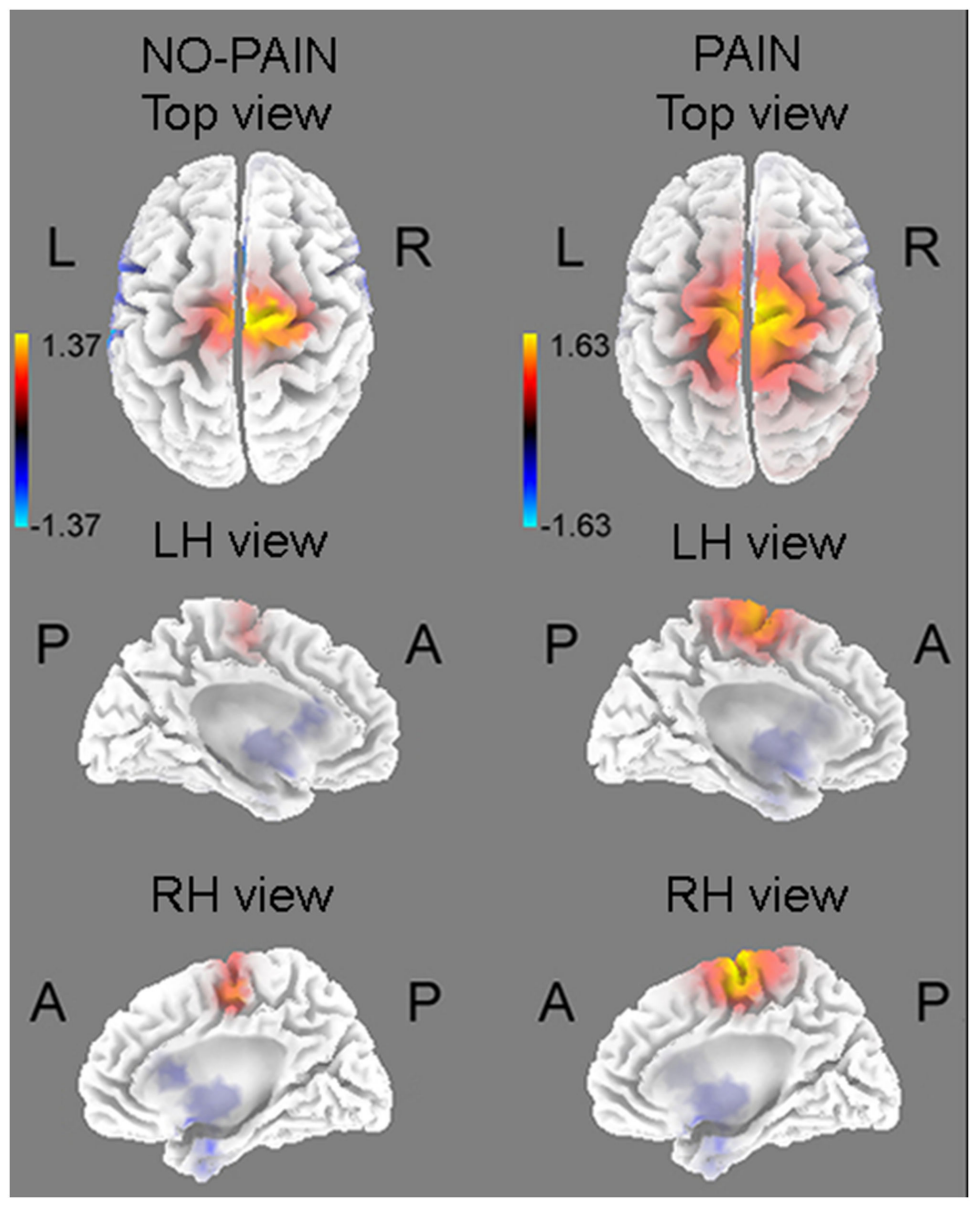
Source localization computed with sLORETA in the 300- to 600-ms epoch: BR group, compared with SC, showed decreased activations of superior frontal gyrus/ACC only in pain condition.

sLORETA analyses carried out on the comparison BR vs. SC groups revealed a significant different activity in the 300-600 ms interval after pain electrical stimulation (t (26) = 1.63, p < 0.05), but not after no-pain pulses (t (26) = 1.37, n.s.). Source analysis located the cortical generator of the 300-600 ms component in the medial portion of superior frontal gyrus/cingulate gyrus, slightly shifted on the right (Brodmann Areas (BAs) 6, 24; coordinates: 5, -5, 63; Figure 4).

Although the between-groups difference was not significant for the no-pain condition, the location of the main generator was similar to that found for pain stimuli (i.e., right superior frontal gyrus/cingulate gyrus; BAs 6,24; coordinates: 10, -11, 54).

### Correlation analyses

**Figure 5:**
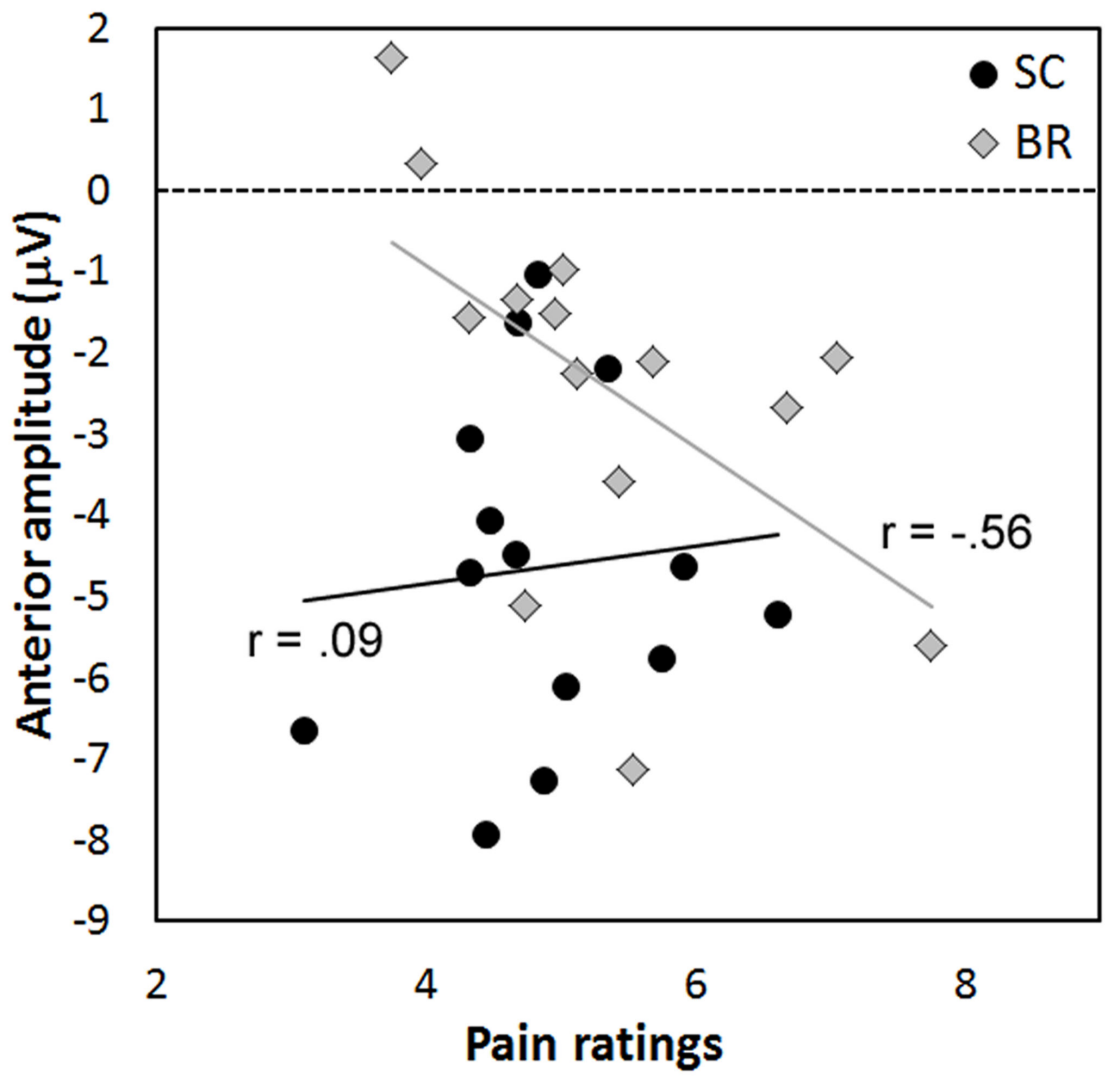
Pearson’s correlations, for the painful condition, between subjective evaluations and the amplitude of the late potentials (300-600-ms epoch) at anterior clusters in the BR and SC groups.

Since the source analysis located the cortical generator of the pain-related LPP component in BAs 6/24, Pearson’s correlation analyses were carried out, separately for each group (SC and BR), between mean subjective evaluations and mean slow wave amplitude (300-600 ms) in the anterior clusters collapsed (AL + AR) only for the pain stimulation. In SC control group correlation was small and not significant (r_12_ = 0.09, ns). By contrast, in the BR group a significant negative correlation was found in anterior clusters (r_12_ = - 0.56, p < 0.05): the higher the pain ratings, the greater was the negativity in frontal sites (Figure 5). 

## Discussion

The present study aimed at investigating the effects of horizontal body position (i.e., Bed Rest, BR) on pain evaluation and ERP responses elicited by electrical stimulation in young and healthy women. Mean electrical current corresponding to participants’ electrical pain threshold was comparable in the two groups, but the subjective pain evaluations collected during the ERP recording revealed reduced subjective sensitivity to no-pain electrical stimuli in the BR group with respect to Sitting Controls (SC). In contrast, pain intensities (corresponding to over-threshold stimuli) were evaluated similarly by the two groups (Figure 1). In a previous study on the effects of Head Down Bed Rest (HDBR), between-groups differences were found for subjective pain evaluations at both threshold and over-threshold conditions [16]. A simple interpretation of this effect is that horizontal BR is probably less effective in modulating subjective pain/perceptual evaluations, compared to a more extreme condition such as HDBR. It can be noticed that the use of our slow pain threshold adaptive procedure which acquaints participant with electrical stimuli, and an ERP task with 180 electrical stimuli, unavoidably induces a substantial pain habituation so that pain sensitivity decreased in the second ERP phase: indeed, the +40% current stimulation condition (corresponding to Pain condition) led, at the end, to an average pain rating of about 5, still painful but clearly habituated.

In the present experiment, compared with controls, BR participants exhibited reduced ERP late amplitudes from 300 to 600 ms, in posterior and anterior regions of interest, regardless of stimulus intensity (Figures 2 and 3). This is in line with previous reports on inhibition of P2 and late potentials induced by the HDBR position [16]. The lack of a significant interaction involving the intensity factor seems to disagree with the source analysis which separately found a significant group difference for the pain condition, and not significant for the no-pain condition. One possible explanation is that the observed cortical inhibition was generalized to both pain and no-pain conditions: in line with prior experiment on HDBR [16] this was partially true, as also painless responses were reduced, although to a less extent with respect to painful condition. A further explanation, which takes account of all results, relies on the statistical characteristics of slow evoked potentials and the two pain conditions. Compared with early evoked potentials, typically characterized by small variances, the slow potentials have larger within- and between-subjects variances. In addition, the two pain conditions differed by a considerable extent of 80% electrical intensity, therefore the strong painful stimulation (pain condition) elicited evoked potentials with high signal-to-noise ratio, while the weak no-pain condition elicited weaker and noisy effects. In omnibus ANOVA analysis this led to a lack of the interactions including Intensity variable. In source location analysis, which includes activity of all electrodes, especially the central ones, the two conditions were necessarily analyzed separately, and this led to a significant effect in the pain condition and a non-significant effect in the no-pain condition.

Notwithstanding the decreased amplitude exhibited by BR participants, the pattern of posterior activation was similar in both groups, since greater positivity was found in right vs. left posterior locations. This finding is in agreement with that found in past studies using somatosensory evoked potentials, in which authors argued that a long latency posterior positivity is found when the experimental task requires to evaluate stimuli with different painful intensity [8] or in complex situations such as painful stimulation during background affective processing [22], and suggests that the late positive component reflects cognitive evaluation and emotional processes. In the present study, the greater right vs. left posterior amplitude was related to the site of stimulation which was contralateral (left forearm) and corresponded to the cortical projection of the analyzed site. Considering anterior sites, SC group exhibited a bilateral activation, whereas the BR group revealed lower negativity on right vs. left locations. Therefore, the greatest between-group difference was found in anterior right region of interest, with BR showing a significant lower negativity/activation than controls. Taken together, electrophysiological results suggest that BR participants had a poor representation of painful stimuli due to an overall inhibition of the fronto-parietal network, especially at the level of right frontal areas.

Correlation analyses carried out between subjective and cortical responses showed significant results for the pain condition only on BR group’s anterior regions. Indeed, BR participants’ self-evaluations were negatively correlated with the mean LPP amplitude on anterior clusters: the higher the pain ratings, the greater the negativity in anterior locations. Thus, anterior negativity reflected pain-related cortical activation, and the high variability of BR cortical amplitude suggests different effects, across BR participants, of horizontal position on the engagement of frontal areas for pain evaluation. Some participants showed reduced pain sensitivity and decreased anterior negative amplitudes, whereas others, who evaluated painful stimulus with higher ratings, exhibited negative amplitudes and greater pain processing which were comparable to those measured in sitting participants.

Consistent with electrode cluster analysis, source analysis carried out with sLORETA revealed between-group differences in the 300-600 ms interval only for pain intensities, controls showing greater activation of superior frontal gyrus/cingulate gyrus. The main generator of the no-pain condition was found in this same location, without significant between-groups differences. The Superior Frontal Gyrus (BA6) is a cortical structure which includes the premotor cortex and the supplementary motor area (SMA), the two main structures involved in movement planning and selection [29]. The activation of these regions could represent the mechanism underlying protective behaviors, such as the automatic tendency to trigger a physical avoidance reaction with motor involvement of the pain-stimulated arm. The Anterior Cingulate Cortex (ACC; BA24) is involved in several cognitive functions, such as attention orienting, cognitive control and motor inhibition [30-32]. In pain contexts, this structure is one of the main generators of the late positive potentials [1]. In addition, past neuroimaging studies showed that different sub-regions of the ACC were related to subjective pain perception and affective-emotional responses [33], as well as sustained attention and phasic orienting to painful stimuli [3,5,34]. These results suggest that the ACC activation may have a key role for the emotional processing of pain, by orienting attention towards painful relevant stimuli and planning adequate motor reaction/inhibition, an interpretation supported by our recent study, in which Head Down Bed Rest induced inhibition of P3 and Slow Positive Potentials elicited by emotional stimuli [35]. Therefore, slow cortical potentials elicited by either aversive painful stimuli and emotional pictures are consistently inhibited by bed rest position. The greater right vs. left posterior activation revealed by ERP analysis in both groups could reflect enhanced attention to painful stimuli delivered to the left side of the body. This result is in agreement with sLORETA analysis although in the latter, the anterior cortical inhibition observed in BR group was more medial and only slightly right-lateralized. Results are also consistent with past studies which found right-hemisphere dominance in pain perception, regardless of the stimulation side [3,36-38]. In line with our results, Symonds et al. [37] identified five active cortical regions in the right hemisphere involved in electrical pain processing, i.e., middle frontal gurus, anterior cingulate, inferior frontal gyrus, medial superior frontal gyri, and inferior parietal lobe. In addition, a general bias towards the right hemisphere has been found in both attentive responses to salient sensory stimuli [39] and negative emotion processing [9]. According to these studies, the right hemisphere plays a key role in pain modulation, in particular through the activation of the right-lateralized attention system, which automatically orients the cognitive resources to the stimulated body area, but it might also involve the cognitive and emotional processing of aversive stimulation. Thus, the decreased activation of superior frontal gyrus/ACC observed in the BR group confirms the inhibitory effects induced by the horizontal position, suggesting that cognitive and emotional resources are reduced and less available for pain processing and coping.

Concerning the putative mechanism which mediates the influence of body position on brain activity, this goes beyond the aims of the present study, but we hypothesize that among all physiological changes (skeletal-muscular, cardiovascular, hormonal, electrolyte redistribution, neurotransmitter, etc.) which are known to vary with posture (see for a review 40), a probable candidate is cardiovascular deconditioning. Indeed, baroreceptor activity changes with cardiovascular unloading induced by horizontal position and, unlike most hormones which change over long times (days), baroreceptors are sensitive to quick postural changes and are able to influence bottom-up cortical arousal and pain responses [23,41]. It is a matter of future studies to investigate the role of baroreceptors in posture-related pain changes.

Summarizing, our data indicate that the altered cortical processing found for pain electrical stimulations in BR women was the direct consequence of our experimental manipulation (i.e., the horizontal position). Correlation results provided evidence in BR participants of an association between different individual responses to pain levels in women’s anterior areas and the subjective evaluations of these painful stimuli. Therefore, in horizontal bed rest, prefrontal negativity represents an important index which is correlated with pain processing, and the horizontal position was able to alter the neurophysiologic functioning of this neural circuit. It should be noticed that our sample included healthy and young women, and that results of our experimental manipulation were achieved after just 90 minutes after participants’ positioning to horizontal position. A limitation of the study is represented by the short time (only 90 min compared with past 4-h durations) and the relatively small sample of subjects which, together with the manipulated horizontal position, probably led to limited effects in perceptual evaluations and early evoked potentials. Further research should be addressed to study the impact of horizontal position on cognitive functioning in elderly adults, with particular attention to pain processing. Indeed, the analysis of the conditions which alter pain is particularly critical for bedridden hospitalized patients, for whom decreased pain sensitivity might lead to delayed diagnosis of fatal medical complications. Bedridden patients usually lie for long time on the bed, they are often elderly and exhibit age-related cognitive decay: thus, future investigations aimed at clarifying bedridden patients’ pain processing could improve their medical treatment.

In conclusion, compared with sitting controls, healthy BR participants showed an overall inhibition of the fronto-parietal network underlying late phases of pain processing, as revealed by reduced anterior and posterior slow wave amplitudes. In addition, the bed rest group exhibited a selective inhibition of medial-right prefrontal structures (superior frontal gyrus/ACC), which have an important role in cognitive, affective and motor aspects of pain processing. Results highlight the effects of short-term horizontal position – the inhibition of cortical responses to painful stimulation in young and healthy women – and suggest important implications for the clinical treatment and diagnosis of medical complications arising in bedridden patients.
